# [^68^Ga]Ga-DOTA-Siglec-9 Detects Pharmacodynamic Changes of FAP-Targeted IL2 Variant Immunotherapy in B16-FAP Melanoma Mice

**DOI:** 10.3389/fimmu.2022.901693

**Published:** 2022-07-06

**Authors:** Riikka Viitanen, Helena Virtanen, Heidi Liljenbäck, Olli Moisio, Xiang-Guo Li, Valeria Nicolini, Marine Richard, Christian Klein, Tapan Nayak, Sirpa Jalkanen, Anne Roivainen

**Affiliations:** ^1^ Turku PET Centre, University of Turku, Turku, Finland; ^2^ Turku Center for Disease Modeling, University of Turku, Turku, Finland; ^3^ InFLAMES Research Flagship Center, University of Turku, Turku, Finland; ^4^ Department of Chemistry, University of Turku, Turku, Finland; ^5^ Roche Pharma Research and Early Development, Roche Innovation Center Zurich, Schlieren, Switzerland; ^6^ Roche Pharma Research and Early Development, Roche Innovation Center Basel, Basel, Switzerland; ^7^ MediCity Research Laboratory, University of Turku, Turku, Finland; ^8^ Turku PET Centre, Turku University Hospital, Turku, Finland

**Keywords:** immunocytokine, melanoma, PET, fibroblast activation protein, interleukin 2 (IL-2)

## Abstract

Vascular adhesion protein-1 (VAP-1) is an inflammation-inducible adhesion molecule, which supports contact between leukocytes and inflamed endothelium. There is evidence that VAP-1 is involved in the recruitment of leukocytes to melanoma tumors. Interleukin-2 (IL-2)-based immunotherapy is an efficient therapy that promotes immune system activity against cancers but is associated with toxicity. In the present study, we evaluated the feasibility of PET/CT imaging using the radiotracer [^68^Ga]Ga-DOTA-Siglec-9, which is targeted to VAP-1, to monitor pharmacodynamic effects of a novel FAP-IL2v immunocytokine (a genetically engineered variant of IL-2 fused with fibroblast activation protein) in the B16-FAP melanoma model. At 9 days after the inoculation of B16-FAP melanoma cells, mice were studied with [^68^Ga]Ga-DOTA-Siglec-9 PET/CT as a baseline measurement. Immediately after baseline imaging, mice were treated with FAP-IL2v or vehicle, and treatment was repeated 3 days later. Subsequent PET/CT imaging was performed 3, 5, and 7 days after baseline imaging. In addition to *in vivo* PET imaging, *ex vivo* autoradiography, histology, and immunofluorescence staining were performed on excised tumors. B16-FAP tumors were clearly detected with [^68^Ga]Ga-DOTA-Siglec-9 PET/CT during the follow-up period, without differences in tumor volume between FAP-IL2v-treated and vehicle-treated groups. Tumor-to-muscle uptake of [^68^Ga]Ga-DOTA-Siglec-9 was significantly higher in the FAP-IL2v-treated group than in the vehicle-treated group 7 days after baseline imaging, and this was confirmed by tumor autoradiography analysis. FAP-IL2v treatment did not affect VAP-1 expression on the tumor vasculature. However, FAP-IL2v treatment increased the number of CD8^+^ T cells and natural killer cells in tumors. The present study showed that [^68^Ga]Ga-DOTA-Siglec-9 can detect B16-FAP tumors and allows monitoring of FAP-IL2v treatment.

## Introduction

Leukocyte trafficking from blood to tissues is a key factor for normal immune responses. Several endothelial adhesion molecules and their counter-receptors mediate a multistep adhesion cascade, which is well understood. However, it is not well known how tumor-infiltrating leukocytes find their way from blood to cancer tissue. One potential candidate for this process is the expression of vascular adhesion protein-1 (VAP-1/AOC3). VAP-1 is an adhesin and enzyme involved in the multistep adhesion between leukocytes and vascular endothelium (1). It mediates direct binding of leukocytes to endothelium. Moreover, *via* its enzymatic activity (especially production of hydrogen peroxidase) it up-regulates other adhesion molecules and thus, it also indirectly has marked contribution to the inflammatory state of the microenvironment. There is prominent evidence that VAP-1 is involved in the accumulation of CD8^+^ T cells and natural killer (NK) cells to high endothelial venules in peripheral lymph nodes and tonsils (2). Indeed, VAP-1 mediates binding of T cells to sinusoidal endothelial cells in hepatocellular carcinoma, and adhesion of tumor-infiltrating lymphocytes, lymphokine-activated killer cells, and NK cells to tumor vasculature (3, 4). We have previously shown that sialic acid-binding immunoglobulin-like lectin 9 (Siglec-9) is a VAP-1 ligand, and that a labeled Siglec-9 motif-containing peptide can be used for PET imaging of inflammation and B16 melanoma tumors (5, 6).

Interleukin 2 (IL-2) is a small cytokine (15-kDa) with pleiotropic effects on the immune system. Predominantly, IL-2 regulates stimulation of growth, proliferation, activation, and differentiation of T lymphocytes (7). Since the early 1990s, high-dose IL-2 therapy has been used to boost anti-tumor immune responses in metastatic melanoma and renal cell carcinoma. However, the therapeutic use of IL-2 is limited due to a short *in vivo* half-life, the preferential expansion of T regulatory cells (Tregs) and significant toxicity such as vascular leaky syndrome (8). IL-2 produces its effects by binding to IL-2 receptors, which consist of α (CD25), β (CD122), and γ (CD132) subunits. Both β and γ subunits are essential for IL-2 binding and signal transduction, whereas the α subunit is not required for IL-2 signaling but is needed for high-affinity binding (9). The heterodimeric IL-2Rβγ is expressed on NK cells, monocytes, and resting CD8^+^ and CD4^+^ T cells (10–12). The heterotrimeric IL-2Rαβγ is constantly expressed on Tregs, and some NK cells and activated T cells can express the IL-2Rα after stimulation by IL-2 (12). The basis of IL-2 anti-tumor activity is the ability to expand and activate effector T cells. However, IL-2 also activates Tregs, in particular CD4^+^CD25^+^FoxP3^+^ T cells, which have immunosuppressive properties (13).

Several strategies have been developed to overcome the harmful side effects of IL-2-based therapy and to achieve better anti-tumor efficacy, e.g., introducing mutations into IL-2 fusion proteins affecting IL-2R subunit binding, and/or targeting IL-2 to tumor cells (14). We have recently designed a novel genetically engineered variant of IL-2 (IL2v), which activates CD8^+^ T cells and NK cells but does not bind to CD25 (15, 16). For better tumor targeting, the IL2v moiety is fused to a fibroblast activation protein (FAP)-specific antibody to selectively promote IL2v immune responses within the tumor microenvironment (16). In the present study, the purpose of the novel FAP-IL2v treatment was to increase the infiltration of pro-inflammatory/cytotoxic immune cells (CD8^+^ T cells, NK cells) into B16 melanoma tumors. We hypothesized that the VAP-1-targeted Siglec-9 tracer can be used to detect immune responses mediated by this novel FAP-IL2v antibody in B16-FAP tumors. We used histology and longitudinal PET/CT imaging using the VAP-1-targeted [^68^Ga]Ga-DOTA-Siglec-9 to monitor the effect of FAP-IL2v treatment in the B16-FAP melanoma model, which comprises B16 cells recombinantly over-expressing murine FAP.

## Materials and Methods

### FAP-IL2v Immunocytokine

FAP-IL2v was produced as murinized molecule based on the high-affinity FAP antibody 4B9, murine IL2v with homologous mutations to human IL2v using a murine IgG1 DAPG (amino acid mutations: replacement of aspartic acid by alanine at position 265, and proline by glycine at position 329) backbone by transient transfection followed by purification using protein A affinity chromatography and size exclusion/ion exchange chromatography. For more details see (16).

### B16-FAP Cell Line

B16 cells were stably transfected with a plasmid for full-length murine FAP. Expression of murine FAP was analyzed by FACS, and stability of expression *in vitro* over 3 months was confirmed. *In vivo* stability of murine FAP expression was confirmed by immunohistochemistry.

### Animals and Study Design

**Figure 1:**
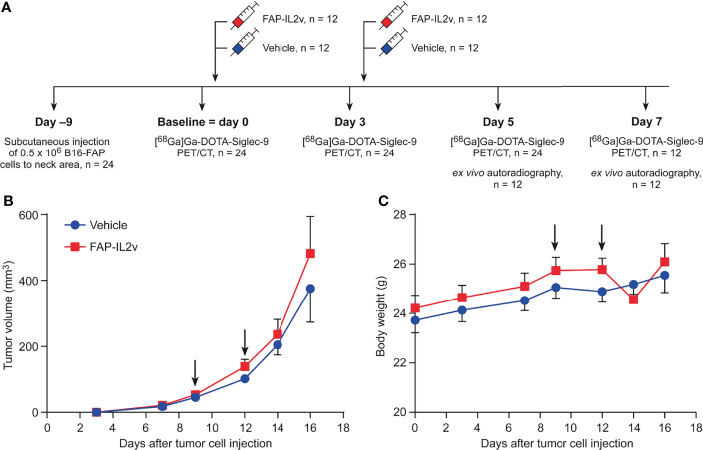
Tumor growth and body weight are not altered after treatment with an IL-2 variant fused to fibroblast activation protein (FAP-IL2v). **(A)** Experimental study design. **(B)** Growth curves of B16-FAP melanoma tumors treated with FAP-IL2v or vehicle during the follow-up period (*n* = 6-12/group). Baseline PET/CT imaging was performed on day 9. **(C)** Body weight of FAP-IL2v-treated and vehicle-treated group during the follow-up period (*n* = 6-12/group). The black arrows indicate the administrations of the drug or vehicle.

B16-FAP murine melanoma cells were cultured in RPMI-1640 medium supplemented with 10% fetal calf serum, 5 mM L-glutamine, penicillin-streptomycin, zeocin, and puromycin. The B16-FAP cell line was kindly provided by Roche Pharma and Early Development Innovation Center, Zurich, Switzerland. Immunocompetent C57BL/6J male mice (*n* = 24) aged 9−11 weeks were used. Mice were housed in standardized conditions, and they had access to water and food *ad libitum*. To generate B16-FAP tumors, 0.5 × 10^6^ cells in phosphate-buffered saline (PBS) were injected subcutaneously into the neck area. Mice were randomized into two groups that received either intravenous (i.v.) injections of FAP-IL2v (20 µg; *n* = 12) or PBS as a vehicle (*n* = 12). At 9 days post-injection, PET/CT imaging was performed as a baseline measurement. Immediately after the baseline imaging, mice were treated with i.v. injections of FAP-IL2v or vehicle, and the injections were repeated 3 days later. Subsequent PET/CT was performed 3, 5, and 7 days after the baseline imaging. On day 5 after the baseline imaging, a subset of the mice (FAP-IL2v *n* = 6 and vehicle *n* = 6) were sacrificed after PET/CT and tumors were subjected to *ex vivo* autoradiography. The same procedure was performed on 7 days after the baseline imaging for the remaining subset of mice. The study design is presented in **Figure 1A**. Tumor growth was measured with external caliper on days 3, 7, 9, 12, 14, and 16 after tumor cell injection. Tumor volume (mm^3^) was determined using the formula 0.5 × length × width^2^.

### Radiochemistry

^68^Ga was obtained from a ^68^Ge/^68^Ga generator (Eckert & Ziegler) by elution with 0.1 M HCl. ^68^Ga eluate was added to a mixture of 2-[4-(2-hydroxyethyl)piperazin-1-yl]ethanesulfonic acid (60 mg) and the precursor compound DOTA-Siglec-9 (6 nmol, 14.5 µg dissolved in water; Peptide Specialty Laboratories GmbH). The reaction mixture was heated at 100°C for 15 minutes. After cooling to approximately room temperature in an ice bath, 1 M NaOH solution was used to adjust the pH to neutral. The molar activity of [^68^Ga]Ga-DOTA-Siglec-9 was 29 ± 9.8 MBq/nmol at the end of synthesis, and the radiochemical purity was >95% throughout the study as determined by reversed-phase radio-HPLC (17).

### *In Vivo* PET/CT Imaging

Mice were anesthetized with isoflurane (4−5% for induction and 1.5−2% for maintenance) and the tail vein was cannulated. A CT scan was performed for anatomical reference and attenuation correction. The mice were scanned using a small-animal PET/CT (Inveon Multimodality, Siemens Medical Solutions) at 9 days post-injection as a baseline measurement, and 3, 5, and 7 days after baseline imaging. The mice were i.v. injected with 9.0 ± 0.94 MBq of [^68^Ga]Ga-DOTA-Siglec-9 *via* the tail vein and a 30 min dynamic PET was performed. PET data acquired in a listmode were iteratively reconstructed with an ordered-subset expectation maximization 3-dimensional algorithm into 6 × 10 s, 4 × 60 s, and 5 × 300 s time frames.

Quantitative PET analysis was performed using Inveon Research Workplace 4.1 software (Siemens Medical Solutions). PET and CT images were automatically superimposed. The regions of interest were defined in the tumor and skeletal muscle based on CT image. The uptake of [^68^Ga]Ga-DOTA-Siglec-9 was reported as mean standardized uptake value.

### *Ex Vivo* Autoradiography

To detect luminal expression of VAP-1, the mice were i.v. administrated anti-VAP-1 monoclonal antibody (clone 7-88, 1 mg/kg diluted in saline) 10 min before being sacrificed (18). After the last PET/CT image was obtained, at 30 minutes post-injection of [^68^Ga]Ga-DOTA-Siglec-9, blood was collected by cardiac puncture and mice were sacrificed by cervical dislocation. Tumors were excised and cut in half longitudinally to prepare cryosections and paraffin-embedded sections. Autoradiography was performed using the previously described method (6) and regions of interest were defined in tumors based on hematoxylin-eosin (HE) staining.

### Histology and Immunofluorescence

The 20 µm tumor cryosections were stained with HE and scanned with a digital slide scanner (Pannoramic 250 Flash, 3DHistech Ltd.). For the detection of luminal VAP-1, the 8 µm tumor cryosections were stained with secondary anti-rat immunoglobulin (**Supplementary Table 1**). The slides were scanned with a digital fluorescent slide scanner (Pannoramic Midi, 3DHistech Ltd.). Images were quantified using ImageJ v.1.48 software (National Institutes of Health).

The subcutaneous tumor, spleen, and lymph node samples were fixed with 10% formalin, embedded in paraffin blocks, cut into 4 µm thickness sections, and mounted on superfrost slides (Leica). To study infiltration of immune cells, slides were immunolabeled with NKp46, CD8 and CD4 primary antibodies and fluorochrome-conjugated secondary antibodies (**Supplementary Table 1**). Scan images were obtained with the Vectra Polaris microscope (Akoya Biosciences) and analyzed with HALO image analysis software (Indica Labs).

### Statistical Analysis

The results are presented as mean ± standard error of the mean (SEM). Statistical analyses were performed using GraphPad Prism software v.7.04 (GraphPad Software Inc.). Normality was examined by a Shapiro-Wilk test. The Student’s *t* test was used for normally distributed data, and the nonparametric Mann-Whitney U test was used for all other experiments. Pearson’s correlation coefficient was calculated between two continuous variables. A *P* value of less than 0.05 was considered statistically significant.

## Results

### Effect of FAP-IL2v Treatment on Tumor Growth and Body Weight

The growth curves of B16-FAP melanoma tumors demonstrated that short-term FAP-IL2v treatment did not inhibit tumor growth (**Figure 1B**). There was no significant difference between FAP-IL2v-treated tumors and vehicle-treated tumors at any time point. Two injections of FAP-IL2v were well-tolerated without any signs of severe adverse effects. There was no difference in body weight between FAP-IL2v-treated or vehicle-treated mice (**Figure 1C**). There was a slight drop in the body weight of FAP-IL2v-treated mice after the administration of the drug, but the body weight normalized within 2 days.

### FAP-IL2v Treatment Increased [^68^Ga]Ga-DOTA-Siglec-9 Uptake in B16-FAP Tumors

**Figure 2:**
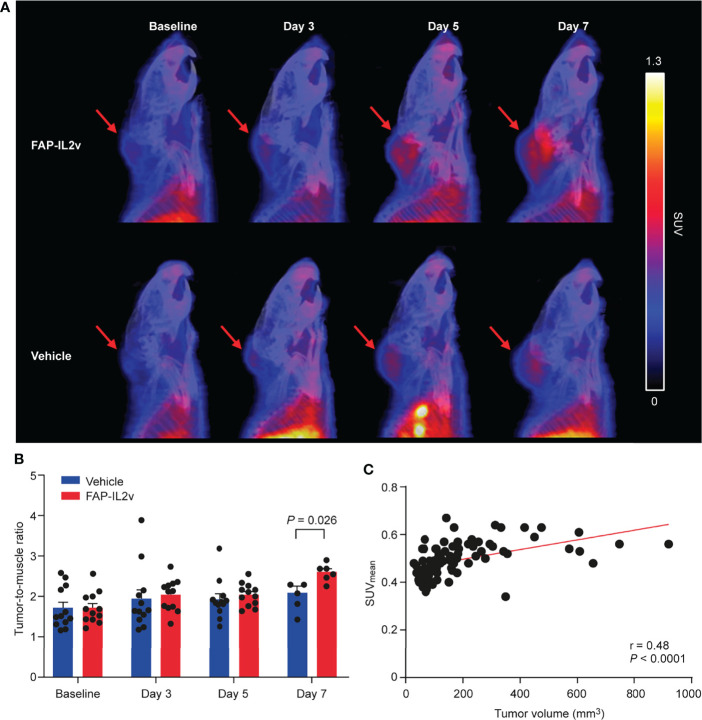
Treatment with an IL-2 variant fused to fibroblast activation protein (FAP-IL2v) enhances [^68^Ga]Ga-DOTA-Siglec-9 uptake in B16-FAP tumors. **(A)** Representative sagittal PET/CT images of FAP-IL2v-treated and vehicle-treated mice at baseline, and 3, 5, and 7 days after baseline imaging. Red arrows indicate B16-FAP melanoma tumors. **(B)** Quantitative analysis of PET/CT images showed that FAP-IL2v treatment increased the tumor-to-muscle ratio of [^68^Ga]Ga-DOTA-Siglec-9 binding (*n* = 6-12/group). **(C)** *In vivo* tumor uptake correlated well with tumor volume during the follow-up period (*n* = 24).

B16-FAP melanoma tumors of the FAP-IL2v-treated and vehicle-treated groups were clearly detected with [^68^Ga]Ga-DOTA-Siglec-9 PET/CT (**Figure 2A** and **Supplementary Figure 1**). Before the treatment, the tumor-to-muscle uptake ratio of [^68^Ga]Ga-DOTA-Siglec-9 was similar in the FAP-IL2v-treated and vehicle-treated groups (**Figure 2B**). Longitudinal [^68^Ga]Ga-DOTA-Siglec-9 PET/CT imaging revealed a relatively equal tumor-to-muscle uptake in the FAP-IL2v-treated and vehicle-treated groups 3 days and 5 days after baseline imaging (**Figure 2B**). However, there was significantly higher tumor-to-muscle ratio of [^68^Ga]Ga-DOTA-Siglec-9 in the FAP-IL2v-treated group than in the vehicle-treated group 7 days after baseline imaging (2.6 ± 0.091 vs. 2.1 ± 0.17, *P* = 0.026). To determine whether the increased uptake of [^68^Ga]Ga-DOTA-Siglec-9 in tumors was associated with increased tumor volume, we performed correlation analysis between *in vivo* PET results from each mouse and individual tumor volumes at various time points. The uptake of [^68^Ga]Ga-DOTA-Siglec-9 into individual B16-FAP tumors correlated significantly with the tumor volume (r = 0.48, *P* < 0.0001) (**Figure 2C**). In B16-FAP tumors, double immunofluorescence staining revealed that VAP-1-positive blood vessels co-localized with the biotinylated Siglec-9 peptide in both the tumor tissue and the tumor periphery (**Supplementary Figure 2** and **Supplementary Figure 3**). Together, these results indicate that [^68^Ga]Ga-DOTA-Siglec-9 binding is enhanced in B16-FAP tumors after FAP-IL2v treatment.

**Figure 3:**
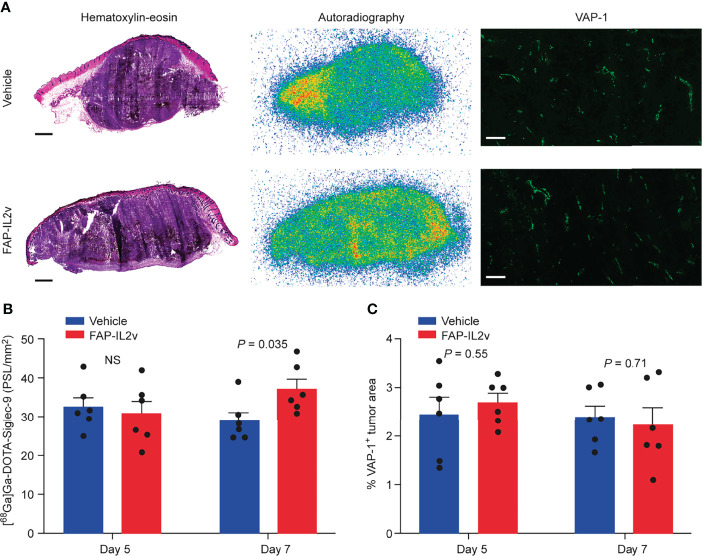
Autoradiography of [^68^Ga]Ga-DOTA-Siglec-9 in B16-FAP tumors. **(A)** Representative B16-FAP tumor sections stained with hematoxylin-eosin (HE), the corresponding autoradiographs and vascular adhesion protein-1 (VAP-1) immunofluorescence staining from mice treated with an IL-2 variant fused to fibroblast activation protein (FAP-IL2v), and vehicle-treated mice 7 days after baseline imaging. The scale bar is 1 mm for the HE-stained image and 200 µm for VAP-1 staining. **(B)** Quantification of tumor autoradiographs showing the distribution of [^68^Ga]Ga-DOTA-Siglec-9 in FAP-IL2v-treated and vehicle-treated mice (*n* = 6/group/timepoint). Results are expressed as photostimulated luminescence per square millimeter (PSL/mm2). NS, not significant. **(C)** The area of VAP-1-positive blood vessels from B16-FAP tumors (*n* = 6/group/timepoint).

PET imaging data were confirmed by *ex vivo* tumor autoradiography analysis. Representative [^68^Ga]Ga-DOTA-Siglec-9 autoradiographs and quantitative results of tumor uptake are presented in **Figures 3A, B**. Autoradiography of B16-FAP tumors 5 days after baseline imaging showed that there was no significant difference in [^68^Ga]Ga-DOTA-Siglec-9 uptake between the FAP-IL2v-treated and vehicle-treated groups (**Figure 3B**). However, the tumor uptake was significantly higher in the FAP-IL2v-treated group than in the vehicle-treated group 7 days after baseline imaging (37 ± 2.6 vs. 29 ± 2.2 PSL/mm^2^, *P* = 0.035). Immunofluorescence staining of B16-FAP tumors revealed that the expression of VAP-1 on tumor vasculature was similar in FAP-IL2v-treated and vehicle-treated groups. Quantification of the VAP-1-positive tumor area showed that FAP-IL2v treatment did not affect the expression of VAP-1-positive blood vessels (**Figure 3C**). Together, these results indicate that [^68^Ga]Ga-DOTA-Siglec-9 can detect increased tumor uptake of FAP-IL2v.

### FAP-IL2v Treatment Alters Immune Cell Populations

**Figure 4:**
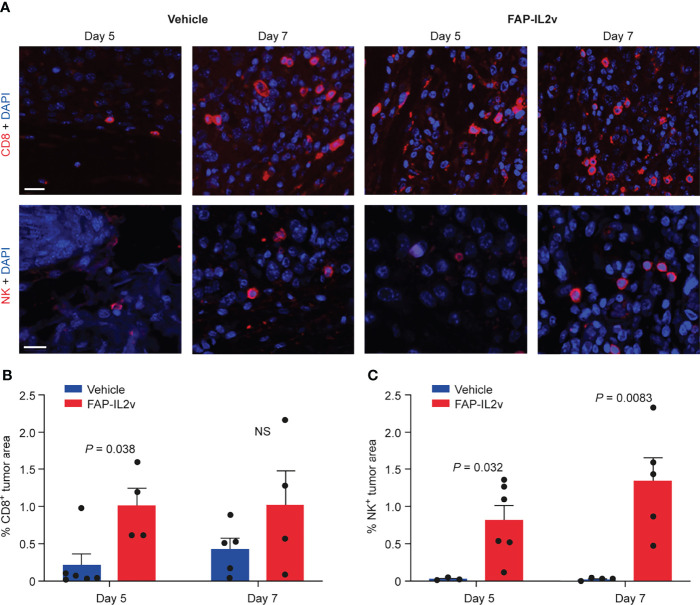
IL-2 variant fused to fibroblast activation protein (FAP-IL2v) treatment increases the number of CD8^+^ T cells and NK^+^ cells in B16-FAP melanoma tumors. **(A)** The expression of CD8^+^ T cells (upper row) and NK^+^ cells (lower row) were visualized in tumor sections 5 and 7 days after baseline imaging in tumors from mice treated with FAP-IL2v and vehicle-treated mice. The scale bar is 20 µm. **(B)** Quantitative analysis of immunofluorescence staining indicates the area of the tumor infiltrated by CD8^+^ T cells (*n* = 4-6/group). NS, not significant. **(C)** Quantitative analysis of immunofluorescence staining indicates the area of the tumor infiltrated by NK^+^ cells (*n* = 3-6/group).

FAP-IL2v treatment induced expansion of CD8^+^ T cells and NK^+^ immune cells in B16-FAP tumors at both time points (**Figure 4A**). There were very few CD8^+^ T cells in the tumors of the vehicle-treated group 5 days after baseline imaging but the number of CD8^+^ T cells slightly increased 7 days after baseline imaging. The number of CD8^+^ T cells in the tumors of the FAP-IL2v-treated group was higher than in the vehicle-treated group and remained constant at both time points. A significant difference in the tumor area infiltrated by CD8^+^ T cells between FAP-IL2v-treated and vehicle-treated groups was detected only at 5 days after baseline imaging (*P* = 0.038; **Figure 4B**). NK cell staining showed that there was a significantly higher infiltration of NK cells into the tumors of the FAP-IL2v-treated group than the vehicle-treated group 5 and 7 days after the baseline imaging (*P* = 0.032 and *P* = 0.0083 respectively; **Figure 4C**). FAP-IL2v treatment did not alter the number of CD4^+^ T cells in tumors compared with the vehicle-treated group, but there was higher increase of CD4^+^ T cells in the tumor on day 7 compared to Day 5, which is consistent with the findings of the tracer (**Supplementary Figure 4**).

**Figure 5:**
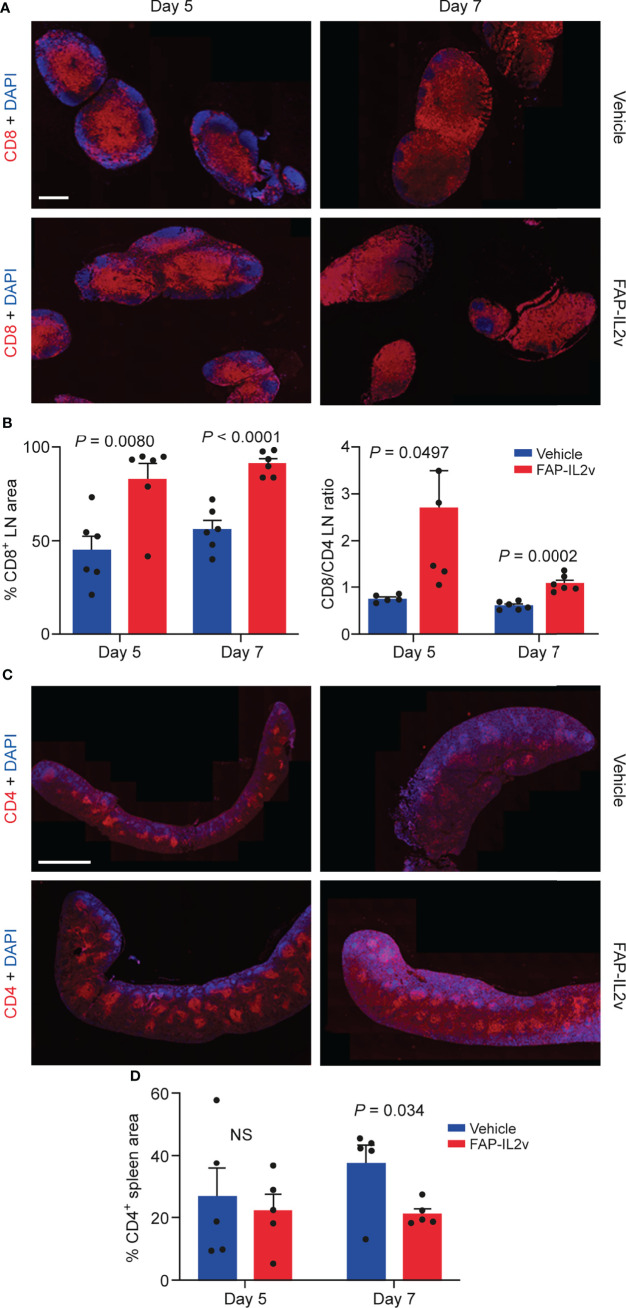
Treatment of mice with an IL-2 variant fused to fibroblast activation protein (FAP-IL2v) induces alterations in immune cell populations. **(A)** Representative immunofluorescence staining of CD8^+^ T cells in lymph node (LN) sections from vehicle-treated and FAP-IL2v-treated mice. The scale bar is 800 µm. **(B)** The area of the LN stained positive for CD8^+^ T cells (as assessed by quantitative analysis of immunofluorescence staining) and the CD8^+^/CD4^+^ ratio in the LN (*n* = 5-6/group). **(C)** Representative immunofluorescence staining of CD4^+^ T cells in spleen sections from FAP-IL2v-treated and vehicle-treated mice. The scale bar is 2 mm. **(D)** Quantitative analysis of immunofluorescence staining indicates the area of the spleen infiltrated by CD4^+^ T cells (*n* = 5/group). NS, not significant.

FAP-IL2v treatment also altered immune cell populations in lymph nodes and spleen (**Figure 5**). In the lymph nodes, FAP-IL2v significantly increased the number of CD8^+^ T cells 5 days (*P* = 0.008) and 7 days after the baseline imaging (*P* < 0.0001; **Figure 5B**). FAP-IL2v treatment did not alter the absolute number of CD4^+^ T cells in lymph nodes but significantly increased the ratio of CD8^+^/CD4^+^ T cells 5 days after the baseline imaging (*P* = 0.0497; **Figure 5B**). The difference in the CD8/CD4 ratio between the groups reduced 7 days after the baseline imaging, but it was still significantly higher in the FAP-IL2v-treated group (*P* = 0.0002). FAP-IL2v treatment did not alter the number of CD4^+^ T cells (**Figure 5C**) in the spleen. FAP-IL2v treatment reduced CD4^+^ T cell staining in the spleen compared with the vehicle-treated group (21.2 ± 1.7% vs. 37.3 ± 6.1%, *P* = 0.034) 7 days after the baseline imaging (**Figure 5D**). There were no differences between groups in splenic CD8^+^ T cells (**Supplementary Figure 5**). No or very few NK^+^ cells were detected in all spleens and lymph nodes (**Supplementary Figure 6**).

## Discussion

In this study, we focused on longitudinal quantitative PET/CT imaging with VAP-1-targeting [^68^Ga]Ga-DOTA-Siglec-9 to assess the pharmacodynamic effect of the novel tumor-targeting FAP-IL2v immunocytokine in mice bearing B16-FAP melanoma tumors. We found that although primary B16-FAP tumor volumes were similar in vehicle- and FAP-IL2v-treated mice, the tumor uptake of [^68^Ga]Ga-DOTA-Siglec-9 was increased in FAP-IL2v-treated mice. We demonstrated a clear correlation between larger tumor volume and higher tracer uptake, which supports the observed pharmacodynamic effects of FAP-IL2v therapy. FAP-IL2v treatment did not alter the number of VAP-1 positive blood vessels in B16-FAP tumors. However, the immunocytokine produced pharmacodynamic changes in immune cell populations in B16-FAP tumors.

A previous study indicated that wild-type IL2 conjugated to tumor-specific antibodies administered as a monotherapy shows poor biodistribution and efficacy in the B16 melanoma tumor microenvironment (19). Generally, the B16 melanoma tumor model is characterized by poor immunogenicity that is relatively resistant to systemic IL2 treatment (20–22). In the current study, the murine B16 melanoma cell line was transfected with FAP to make tumors more likely to elicit an immune response. Under normal physiological conditions, tissue expression of FAP is very low, yet expression increases during wound healing (23). However, FAP is expressed on the surface of carcinoma-associated fibroblasts in the tumor stroma in more than 90% of solid tumors including breast, colorectal, skin, and pancreatic cancers, and in some soft tissue sarcomas (24, 25). Since IL-2 based immunotherapy is efficient against metastatic melanomas, albeit associated with toxicity (8), we designed a tumor-targeting FAP-IL2v with abolished CD25 binding. Our results indicate that FAP-targeted IL2v treatment induced strong local expansion of CD8^+^ T cells and NK cells at tumors. A carcinoembryonic antigen-targeted IL2 variant produced a similar effect on immune cell populations (15). In addition, FAP-IL2v activated a systemic effect by increasing the number of CD8^+^ T cells in the lymph nodes. One explanation for this could be that although very low there is still some expression of FAP in the lymph nodes and because there are more CD8+ cells in this organ at baseline, the observed expansion may be higher. The consequences of systemic infiltration or more pronounced expansion of immune cells in the periphery is IL2 related toxicity including immune/cytokine storm, vascular leak syndrome and lung edema. With the FAP-IL2v, this peripheral effect is much lower compared to proleukin, but it is not completely abolished. Thus, there is still a dose limiting shortcoming for this drug, albeit not as extreme as with proleukin.

Studies have shown that IL2 variants can reduce tumor size (15, 26). However, these responses were either seen in inflamed models such as MC38 or PancO2, or when IL2 variants were used as part of combination therapy in the B16 model (22, 27). In our study, twice-injected FAP-IL2v treatment as monotherapy did not inhibit the growth of B16-FAP tumors. This finding may be due to our experimental protocol, which used short-term treatment with FAP-IL2v and a short follow-up period. Recent studies support this claim; FAP-IL2v used in combination with another drug mediates anti-tumor activity (16, 28). This is in the line that B16 does not respond to IL-2 treatment alone, but needs to be combined (22, 27). Thus, it was not expected that FAP-IL2v would work in B16. During immunotherapy, pseudoprogression of disease may be observed, whereby the response to treatment first causes an increase in tumor volume due to infiltration of immune cells. This phenomenon has been observed with immune checkpoint inhibitors in melanoma and other solid tumors (29). However, to show efficacy FAP-IL2v would need to be combined with a checkpoint inhibitor and an agent that results in antigen release to boost the level of antigen presenting cells. Studies in which infiltration of inflammatory cells induces a decrease in tumor volume, which is an important endpoint in treatment monitoring, would further confirm the importance of monitoring the pharmacodynamics of immune cell populations.

In the subcutaneous B16-FAP melanoma model, we found that FAP-IL2v significantly increased the tumor uptake of VAP-1-targeted [^68^Ga]Ga-DOTA-Siglec-9 7 days after baseline imaging, as measured by *in vivo* PET and *ex vivo* autoradiography methods. Despite increased [^68^Ga]Ga-DOTA-Siglec-9 uptake, FAP-IL2v did not change the density of VAP-1-positive blood vessels in tumors. We know that mutations at glycosylation sites on the surface of the VAP-1 molecule reduce lymphocyte adhesion to endothelium and affect the catalytic activity of VAP-1/AOC3 (30). Based on this finding, treatment with FAP-IL2v may alter the glycosylation of VAP-1 and affect the binding of [^68^Ga]Ga-DOTA-Siglec-9 to the enzymatic groove of VAP-1. Previously, using a mouse B16 melanoma model, we demonstrated that [^68^Ga]Ga-DOTA-Siglec-9 can be used to image tumor-associated inflammation. In that study, [^68^Ga]Ga-DOTA-Siglec-9 could detect subcutaneous B16 melanoma tumors 7 days and 9 days after tumor cell inoculation (6). Moreover, blood vessels within melanoma tumors express VAP-1 in humans and mice (31, 32). In head and neck squamous cell carcinoma, VAP-1 mediates the binding of tumor-infiltrating lymphocytes and NK cells to tumor endothelium (4). In line with these findings, our results further demonstrate that [^68^Ga]Ga-DOTA-Siglec-9 can be used to reflect tumor-associated inflammation. In addition to melanoma, this tracer could be used to image various other types of cancers. However, in clinical settings it will be wise to evaluate VAP-1 expression in tumor biopsies before the use of the tracer, if possible. This will be easily performed as there are several nicely working antibodies against VAP-1.

## Conclusion

In a B16-FAP melanoma model, we found that FAP-IL2v treatment increased CD8^+^ T cell and NK cell infiltration, which may partly explain the increased tumor uptake of VAP-1-targeted [^68^Ga]Ga-DOTA-Siglec-9. The pharmacodynamic effects of FAP-IL2v-mediated immunotherapy, assessed by PET and immunofluorescence, were observed even in the absence of tumor shrinkage. These findings suggest that [^68^Ga]Ga-DOTA-Siglec-9 is a potential imaging agent for *in vivo* imaging of tumor-associated inflammation and provides a tool for monitoring cancer immunotherapy.

## Data Availability Statement

The datasets generated during and/or analyzed during the current study are available from the corresponding author on reasonable request.

## Ethics Statement

The animal study was reviewed and approved by The National Project Authorization Board in Finland. (Licence numbers ESAVI/3116/4.10.07/2017 and ESAVI/5239/04.10.07/2017)

## Author Contributions

Conception and design: RV, HV, CK, TN, SJ, and AR; analysis and interpretation of data: RV, HV, HL, OM, X-GL, VN, MR, CK, TN, SJ, and AR; drafting of the manuscript: RV, CK, TN, SJ, and AR; revising it critically for important intellectual content: RV, HV, HL, OM, X-GL, VN, MR, CK, TN, SJ, and AR. All authors contributed to the article and approved the submitted version.

## Funding

This study was financially supported by grants from the Academy of Finland (#258814), the Jane and Aatos Erkko Foundation, the State Research Funding of Turku University Hospital, the Finnish Cultural Foundation, the Instrumentarium Science Foundation, and the Drug Research Doctoral Programme of the University of Turku Graduate School.

## Conflict of Interest

SJ owns stock in Faron Pharmaceuticals. CK, VN, and MR declare employment, patents and ownership interest with Roche, and TN was employed by Roche at the time of the study.

The remaining authors declare that the research was conducted in the absence of any commercial or financial relationships that could be construed as a potential conflict of interest.

## Publisher’s Note

All claims expressed in this article are solely those of the authors and do not necessarily represent those of their affiliated organizations, or those of the publisher, the editors and the reviewers. Any product that may be evaluated in this article, or claim that may be made by its manufacturer, is not guaranteed or endorsed by the publisher.
