# Supplementary material for: [68Ga]Ga-DOTA-Siglec-9 Detects Pharmacodynamic Changes of FAP-Targeted IL2 Variant Immunotherapy in B16-FAP Melanoma Mice
Source: Front Immunol. 2022 Jul 6;13:901693. doi: 10.3389/fimmu.2022.901693 (PMC9298541; doi:10.3389/fimmu.2022.901693)
Supplement: Supplementary file 1 [file DataSheet_1.pdf]

Supplementary Material

1 Supplementary methods

1.1 Double immunofluorescence staining for VAP-1 and Siglec-9 peptide in B16-FAP tumors

To study the co-localization of VAP-1 and binding of Siglec-9 motif peptide, mouse B16-FAP tumor cryosections were double-stained. For detection of VAP-1, cryosections were incubated with anti-rat-546 (5 µg/mL; Invitrogen A11081) secondary antibody (the mice had been treated with anti-VAP-1 antibody just before the sacrifice). After washing, sections were incubated with biotinylated Siglec-9 peptide (0.28 µg/mL; NeoMPS C17-K(Biotin)-NH_2 cyclic) followed by streptavidin-Alexa Fluor 488 (10 µg/mL; Invitrogen S11223).

2 Supplementary figures and tables

2.1 Supplementary figures

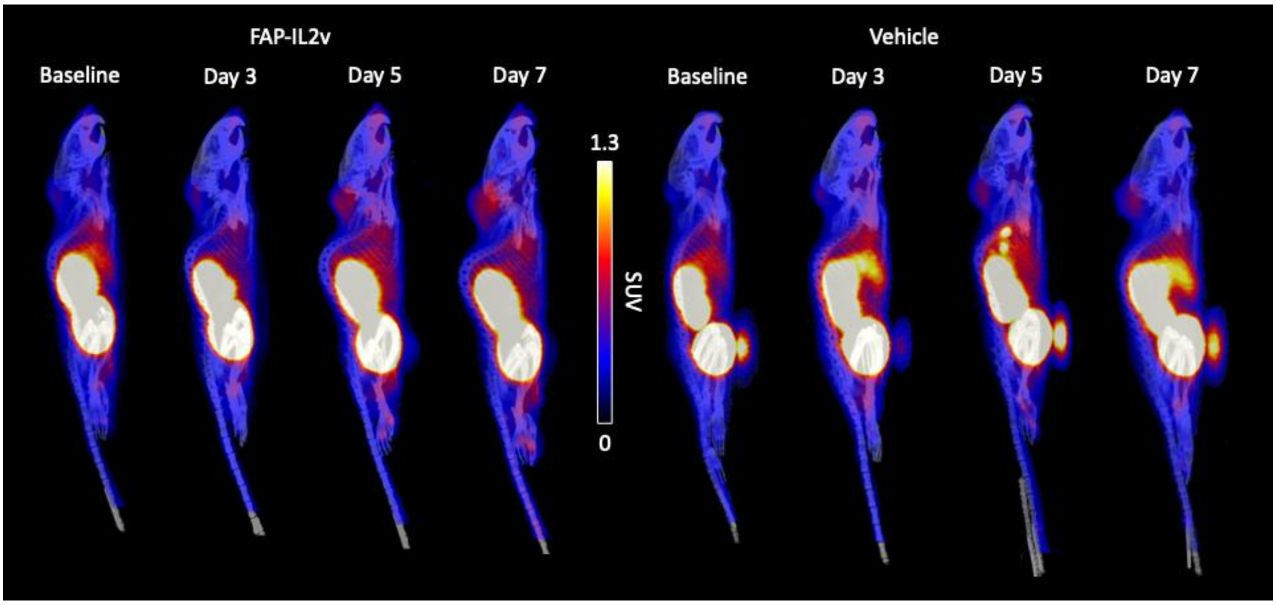

Supplementary Figure 1. Representative whole-body sagittal PET/CT images of FAP-IL2v-treated and vehicle-treated mice at baseline, and 3, 5, and 7 days after baseline imaging. SUV, standardized uptake value.

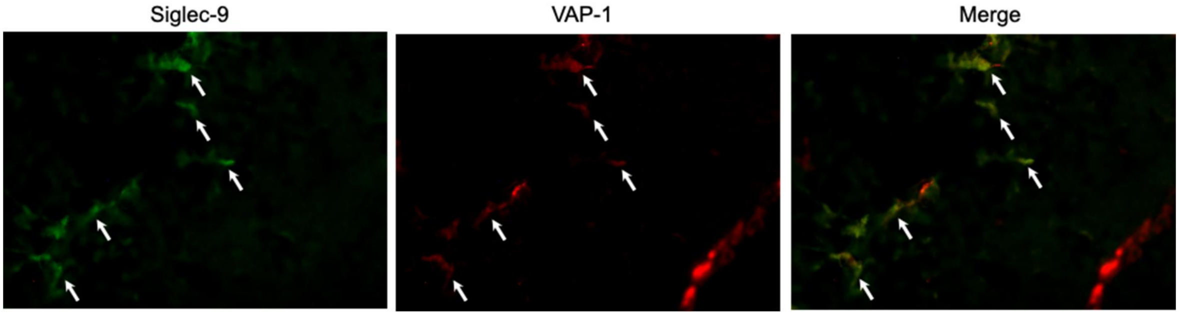

Supplementary Figure 2. Double immunofluorescence staining for Siglec-9 peptide and VAP-1 in B16-FAP tumors. Left panel, binding of biotinylated Siglec-9 peptide (green). Middle panel, VAP-1-positive blood vessels detected by fluorescein-conjugated anti-VAP-1 antibody (red). Right panel, merge figure. White arrowheads indicate double-positive blood vessels.

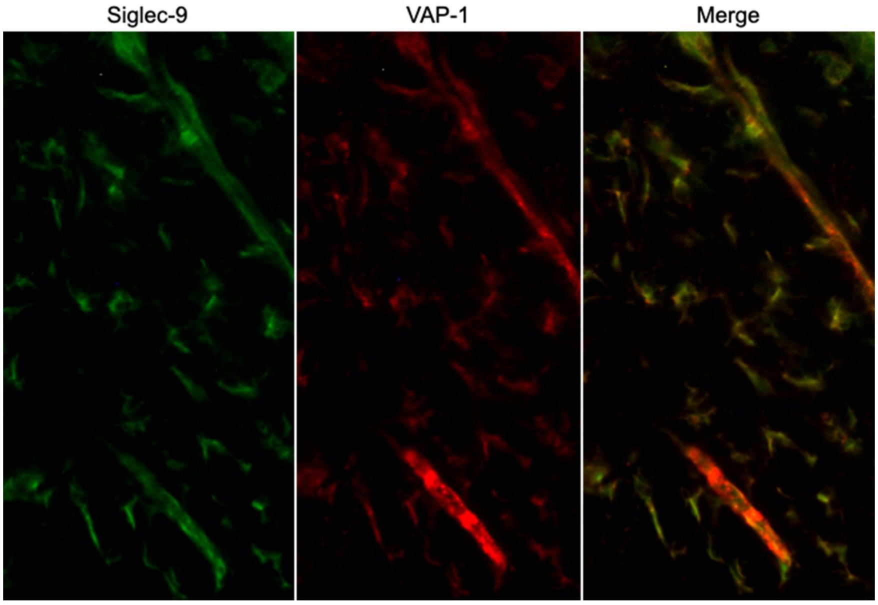

Supplementary Figure 3. Double immunofluorescence staining for Siglec-9 peptide and VAP-1 in the B16-FAP tumor periphery. Left panel, binding of biotinylated Siglec-9 peptide (green). Middle panel, VAP-1-positive blood vessels detected by fluorescein-conjugated anti-VAP-1 antibody (red). Right panel, merge figure.

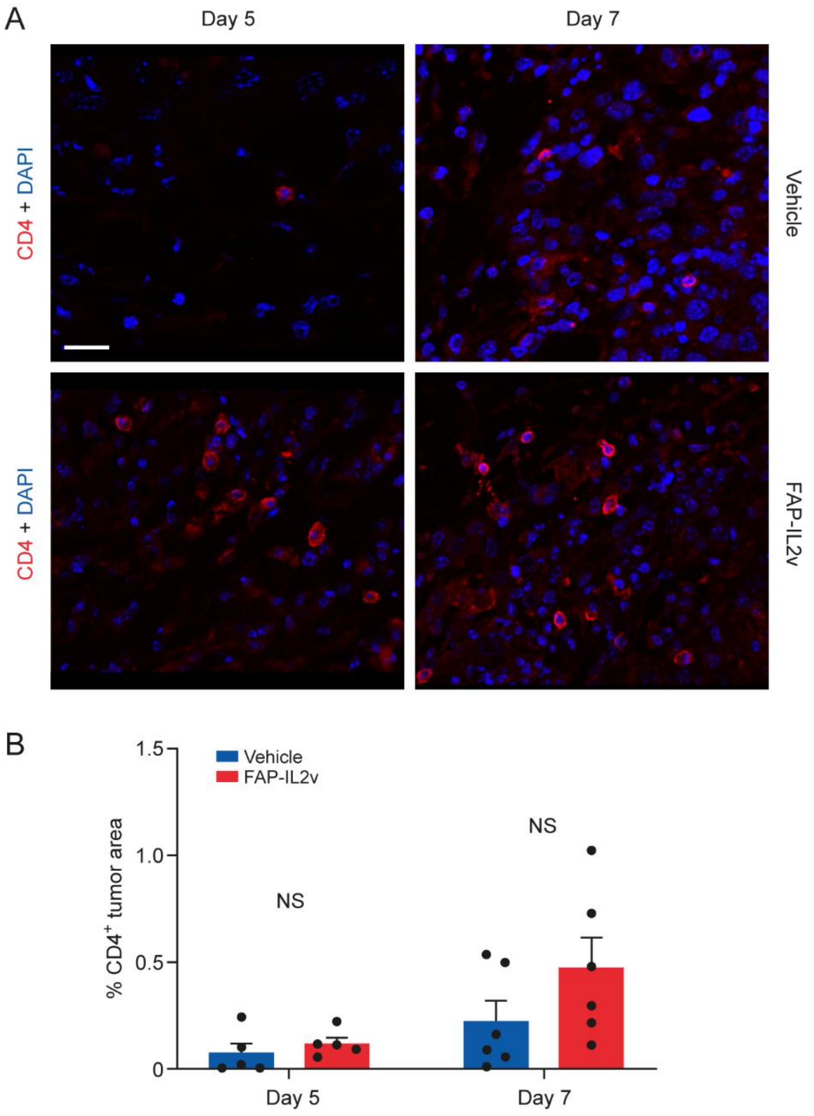

Supplementary Figure 4. (A) Representative immunofluorescence staining of CD4$^+$ T cells in B16-FAP tumor sections from FAP-IL2v-treated and vehicle-treated mice. The scale bar is 20 µm. **(B)** Quantitative analysis of immunofluorescence staining indicates the area of the tumor infiltrated by CD4$^+$ T cells (n = 5–6/group). NS, not significant.

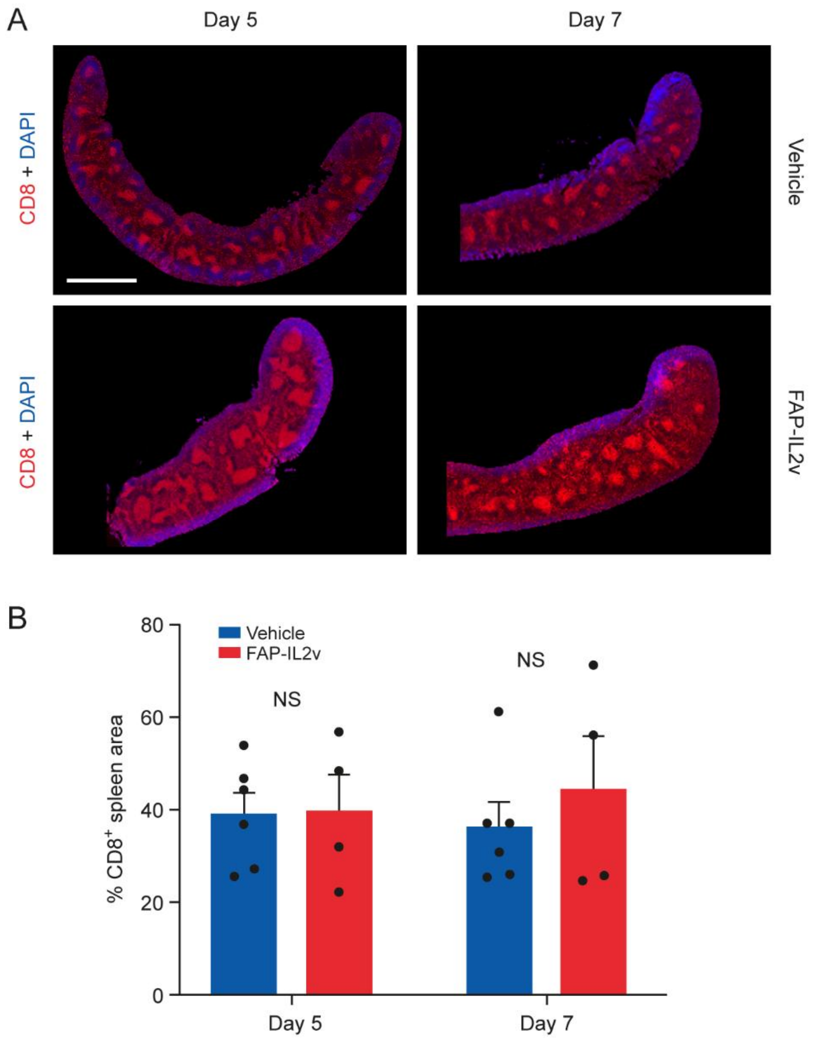

Supplementary Figure 5. (A) Representative immunofluorescence staining of CD8^{+} T cells in spleen sections from FAP-IL2v-treated and vehicle-treated mice. The scale bar is 20 µm. **(B)** Quantitative analysis of immunofluorescence staining indicates the area of the spleen infiltrated by CD8^{+} T cells (n = 4–6/group). NS, not significant.

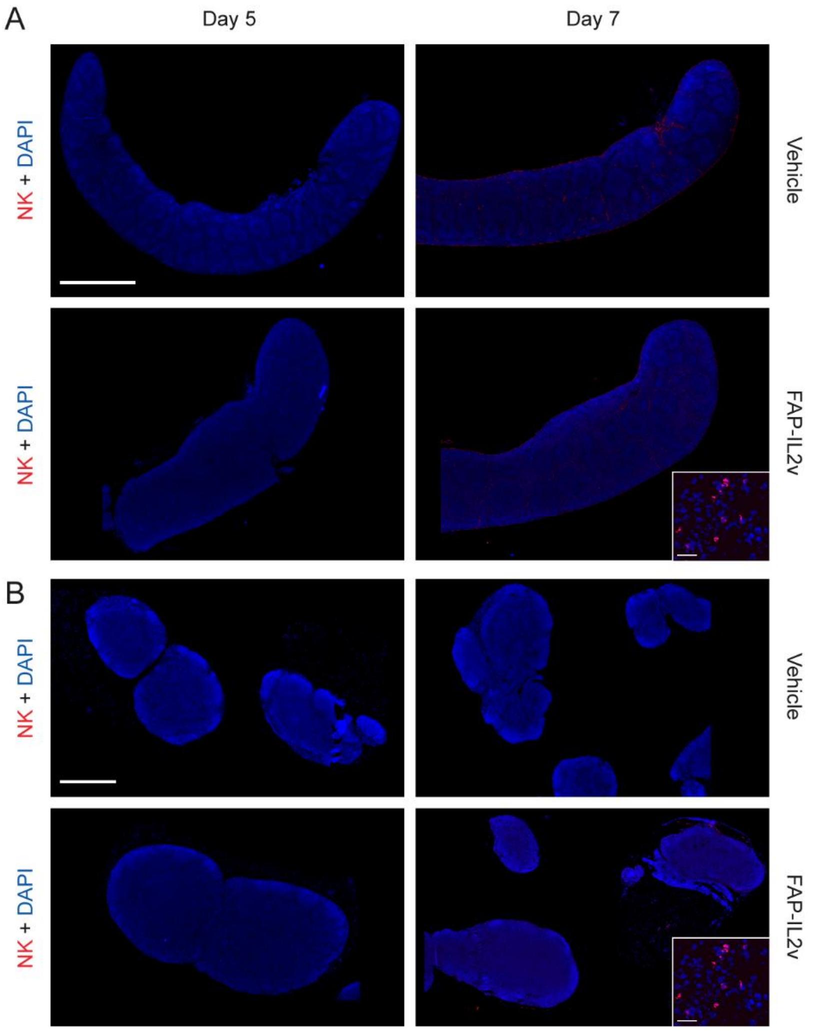

Supplementary Figure 6. Representative immunofluorescence staining of NK^{+} cells in **(A)** spleen and **(B)** lymph node sections from FAP-IL2v-treated and vehicle-treated mice. No or very few NK^{+} cells were detected in all spleens and lymph nodes at both time points, so no quantitative analysis was performed. The scale bar in (A) is 2 mm for spleen and 20 µm for positive control. Scale in (B) is 800 µm for lymph node and 20 µm for positive control.

Supplementary Table 1 Primary antibodies and detection methods used for immunofluorescence staining

Antibody	Clone	Dose	Dilution	Manufacturer	Detection
VAP-1	Rat monoclonal anti-mouse VAP-1, 7-88	i.v. 1 mg/kg in saline		Uncommercial, Sirpa Jalkanen´s laboratory	Goat anti-rat IgG Alexa Fluor 488; Invitrogen, A11006
NKp46	Goat polyclonal anti-mouse NKp46, AF2225		1:50	R&D Systems	Discovery Cy5 detection kit; Ventana Medical Systems, VENTANA
CD8	Rat monoclonal anti-mouse CD8, 4SM15		1:300	Thermo Fisher Scientific	Donkey anti-rat IgG Alexa Fluor 647; Jackson ImmunoResearch Laboratories, 712-605-153
CD4	Rat monoclonal anti-mouse CD4, 4SM95		1:500	Thermo Fisher Scientific	Donkey anti-rat IgG Alexa Fluor 647; Jackson ImmunoResearch Laboratories, 712-605-153
					Mounting medium: Prolong Gold antifade reagent with DAPI; Invitrogen, P36935

VAP-1, vascular adhesion protein-1; NKp46, natural killer cell marker; CD8, T cell marker; CD4, T cell marker; i.v., intravenously
